# TDP-43 regulation of stress granule dynamics in neurodegenerative disease-relevant cell types

**DOI:** 10.1038/s41598-018-25767-0

**Published:** 2018-05-15

**Authors:** Yousra Khalfallah, Rachel Kuta, Camille Grasmuck, Alexandre Prat, Heather D. Durham, Christine Vande Velde

**Affiliations:** 1 Department of Biochemistry and Molecular Medicine, Universite de Montreal, Montreal, QC Canada; 2 Department of Neurosciences, Universite de Montreal, Montreal, QC Canada; 3 Centre Hospitalier de l’Universite de Montreal (CHUM) Research Center, Montreal, QC Canada; 4 Montreal Neurological Institute and Department of Neurology/Neurosurgery, McGill University, Montreal, QC Canada

## Abstract

Stress granules (SGs) are cytoplasmic foci that form in response to various external stimuli and are essential to cell survival following stress. SGs are studied in several diseases, including ALS and FTD, which involve the degeneration of motor and cortical neurons, respectively, and are now realized to be linked pathogenically by TDP-43, originally discovered as a component of ubiquitin-positive aggregates within patients’ neurons and some glial cells. So far, studies to undercover the role of TDP-43 in SGs have used primarily transformed cell lines, and thus rely on the extrapolation of the mechanisms to cell types affected in ALS/FTD, potentially masking cell specific effects. Here, we investigate SG dynamics in primary motor and cortical neurons as well as astrocytes. Our data suggest a cell and stress specificity and demonstrate a requirement for TDP-43 for efficient SG dynamics. In addition, based on our *in vitro* approach, our data suggest that aging may be an important modifier of SG dynamics which could have relevance to the initiation and/or progression of age-related neurodegenerative diseases.

## Introduction

TAR DNA-binding protein 43 (TDP-43) was originally characterized as a transcriptional repressor of the HIV-1 genome via binding to the trans-activation response (TAR) element^1^. TDP-43 is a highly conserved, ubiquitously expressed RNA-binding protein associated with the heterogeneous ribonucleoprotein (hnRNP) family. The RNA-binding ability of TDP-43 is conferred by two RNA recognition motifs (RRM1 and RRM2), while the C-terminal glycine-rich region mediates protein-protein interactions. In addition to known roles in alternative splicing and transcriptional regulation in the nucleus^2–4^, TDP-43 functions to stabilize and transport mRNA in the cytoplasm^5,6^. Additionally, TDP-43 is recruited to cytoplasmic RNA granules that are formed following exposure to various environmental stresses (oxidative, osmotic, heat shock, viral infection). These granules, termed stress granules (SGs), are membrane-less organelles that are believed to facilitate cell survival via the storage of non-essential mRNAs, translation factors and RNA-binding proteins during stress exposure^7–9^. SGs follow a linear dynamic featuring an initial nucleation/formation followed by assembly into larger structures, and eventual disassembly as the cell recovers. In transformed cell lines, depletion of TDP-43 has a negative impact on each of these steps^10,11^, indicating a key role for TDP-43 in the regulation of this essential cell survival mechanism.

The term “TDP-43 proteinopathies” first emerged with the discovery of ubiquitinated cytoplasmic inclusions of TDP-43 in the neurons of patients affected with frontotemporal dementia (FTD) and amyotrophic lateral sclerosis (ALS)^12,13^. These diseases impact particular neuronal populations, with ALS being due to motor neuron degeneration and accompanying gliosis^14–16^ and FTD involving extensive degeneration of cortical neurons. Despite TDP-43-containing cytosolic inclusions in neurons and some glial cells being a pathological hallmark of ALS and FTD, the mechanism(s) by which TDP-43 contributes to neurodegeneration remains unclear. Furthermore, a persistent question in the field is whether TDP-43 evokes toxicity via the gain of an unknown cytosolic function or via the loss of some nuclear function. This outstanding question stems from the observation that neurons presenting cytoplasmic pathological inclusions usually also demonstrate a depletion of the nuclear pool of TDP-43^12,13,17^.

Stress response mechanisms are heavily studied in neurodegenerative diseases and there has been a recent convergence on the involvement of SGs in ALS and FTD. Although it is appreciated that SG morphology and composition differ according to the stress stimuli and the cell type^9,18^, few studies have been conducted in cell types relevant to neurodegenerative disease^19^. Thus, we aimed to investigate SG dynamics and the impact of TDP-43 on these structures in primary neurons and glia. We observed distinct differences in SG morphology and dynamics between cortical and motor neurons, astrocytes, and fibroblasts. The data demonstrate that TDP-43 is required for optimal SG dynamics in primary neurons and glia exposed to oxidative stress. Moreover, the dependence on TDP-43 for SG formation was exacerbated in the context of hyperosmotic stress. Finally, neurons “aged” via prolonged culture times had impaired SG dynamics accompanied by decreased TDP-43 expression.

## Results

### Variability in stress granule morphology and dynamics in primary cells

**Figure 1:**
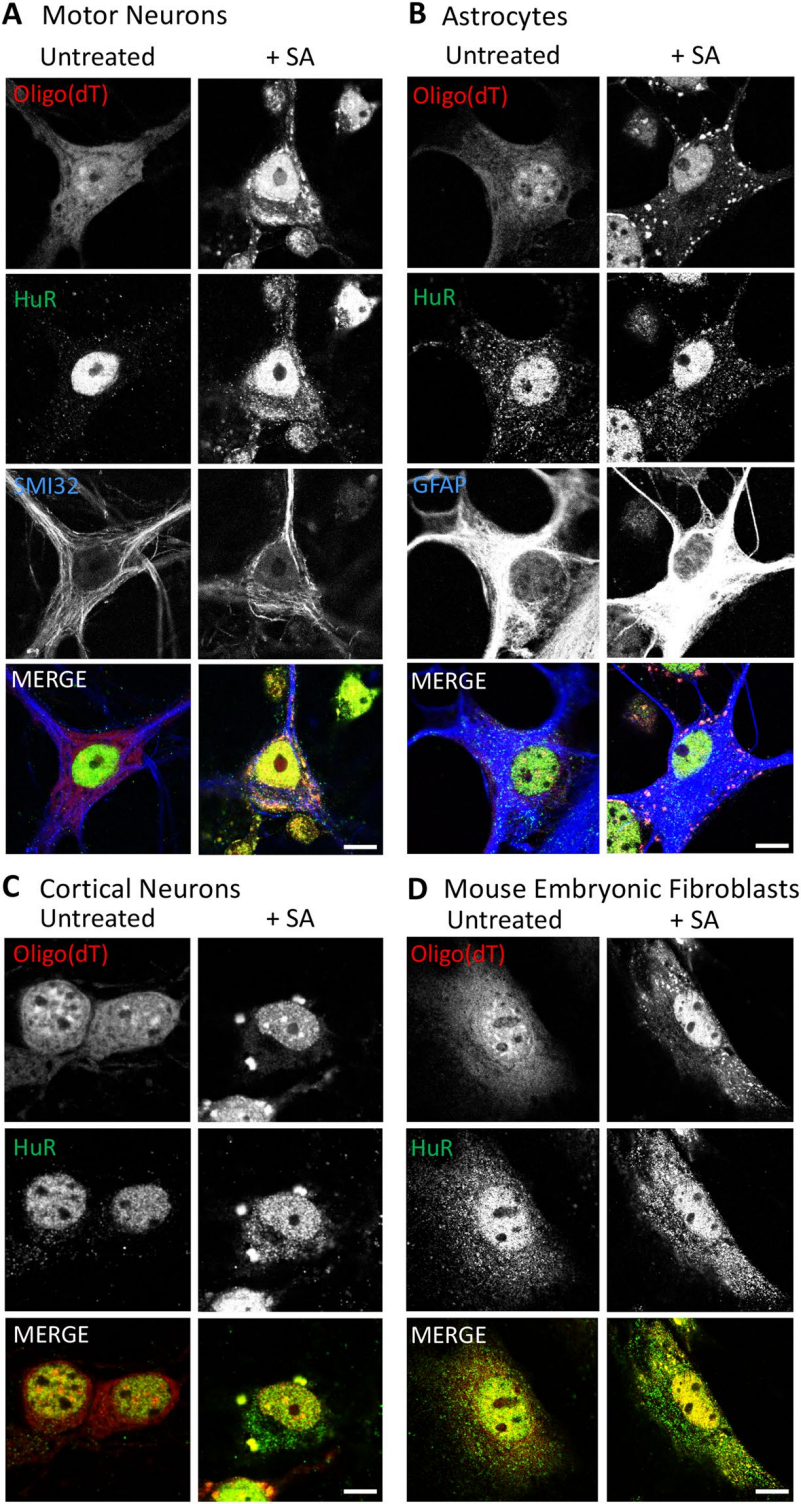
Differences in SG morphology amongst cell types. Primary cultures of (**A)** motor neurons, (**B**) astrocytes, (**C**) cortical neurons and (**D**) mouse embryonic fibroblasts treated (or not) with 0.5 mM of sodium arsenite (+SA). Cytoplasmic SGs were co-labelled with an oligo(dT) probe to track polyadenylated mRNA, and an antibody against HuR (a known SG marker, that can also serve as a nuclear marker by its presence in the nucleus). Note, motor and cortical neurons were treated for 60 min while astrocytes and fibroblasts were treated for 30 min. Scale bar, 10 µm.

To study differences in SG dynamics between cell types, we chose primary cultures of mouse cortical and motor neurons as neuronal subtypes implicated in the spectrum of TDP-43 proteinopathies, especially FTD and ALS^12,20^; and astrocytes, glia cells implicated in ALS progression^14^. As a cell type unrelated to these disease conditions, we also cultured mouse embryonic fibroblasts. Given that the majority of published studies on SGs utilize sodium arsenite (SA) as the SG-provoking agent^21^, our first goal was to determine if SA can provoke SG formation in the primary cultures selected. SGs were labelled with an oligo(dT) probe since polyadenylated mRNA is obligatorily recruited into SGs following stress exposure regardless of cell type, whereas protein composition can vary between cell types, as demonstrated by the variable colocalization of known SG markers HuR^22^ (Fig. 1) and CAPRIN1^23^ (Suppl. Figure 1) with oligo(dT) labelled mRNA. In all of the primary cells examined, SGs were observed following 0.5 mM SA treatment, the standard concentration used in SG experiments^21^, albeit qualitative differences in SG shape and size were noted (Fig. 1, Suppl. Figure 1). Specifically, SGs were easily visible and loosely distributed in a perinuclear fashion in SA-treated motor and cortical neurons (Fig. 1A,C; Suppl. Figure 1A,C). In contrast, SGs in astrocytes were more localized at the cell periphery (Fig. 1B; Suppl. Figure 1B) and SGs in mouse embryonic fibroblasts (MEFs) were frequently smaller and randomly dispersed throughout the cytoplasm (Fig. 1D; Suppl. Figure 1D). These qualitative observations of SGs in astrocytes and MEFs were equivalent at a longer SA exposure time (1 hr), although cell viability was compromised (Suppl. Figure 2). These observations suggest cell type-dependent difference in SG dynamics, which prompted a more detailed examination over time.

**Figure 2:**
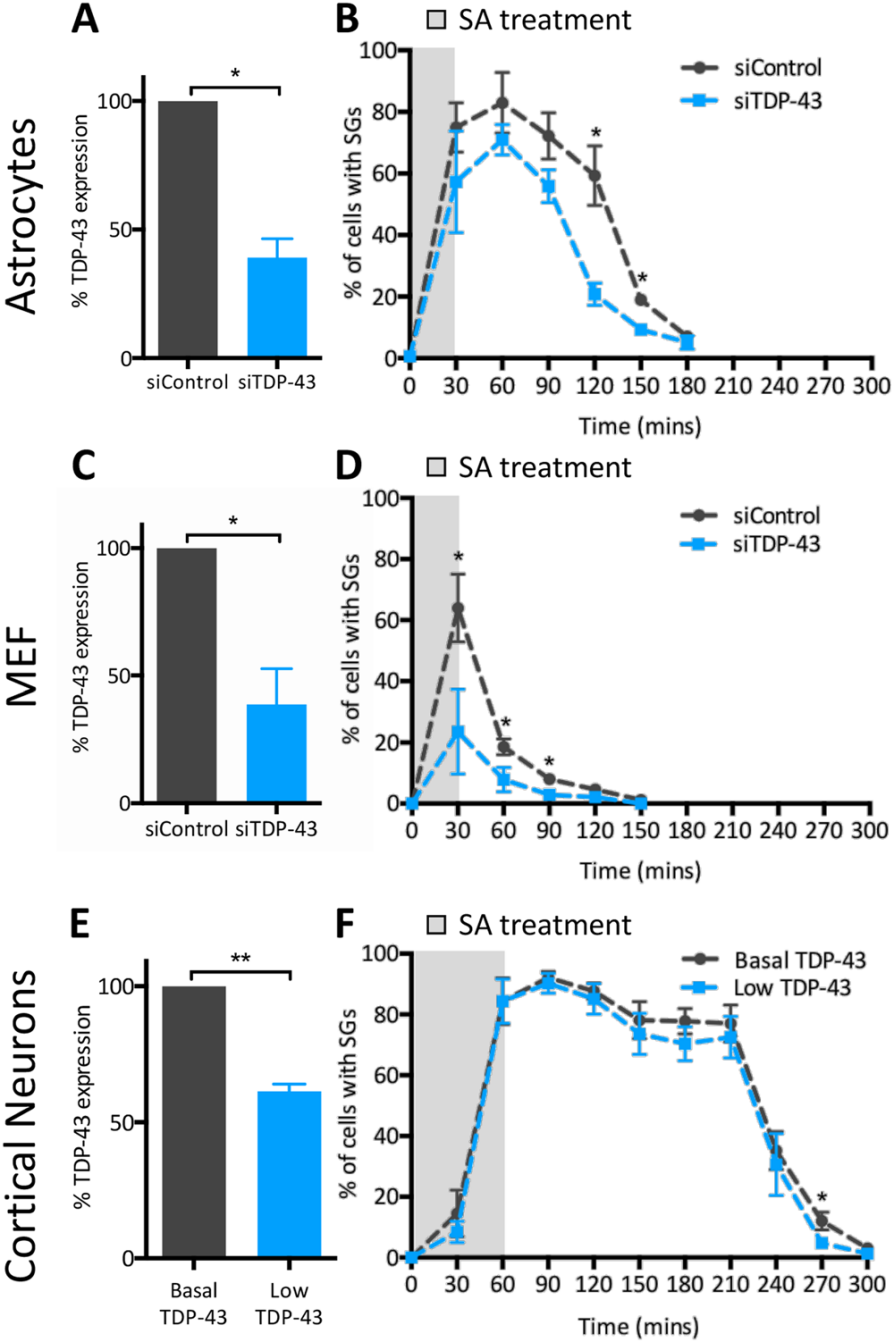
SG kinetics vary according to cell type and are modulated by TDP-43. (**A**,**B**) Primary astrocytes (n = 3, average N = 100 per time point), (**C**,**D**) mouse embryonic fibroblasts (MEF) (n = 3, average N = 100 per time point), and (**E,F**) cortical neurons (n = 4, average N = 70 per time point) were transfected with siRNA (siTDP-43 #1) and subjected to 0.5 mM SA. (A, C, E) TDP-43 expression levels expressed relative to control cultures, as determined by measurement of TDP-43 signal intensity. (**B**,**D**,**F**) Percentage of cells displaying SGs at different time points following SA exposure. Data of 3–4 independent experiments are expressed as the mean ± SEM; Student *t* test *p < 0.05.

In transformed cell lines exposed to 0.5 mM SA, several small SGs assemble within minutes of exposure and then coalesce into a fewer number of larger puncta over a defined time window^10^. In primary astrocytes, a similar pattern was observed in that more than 70% of cells contained SGs following a brief 30 min SA exposure; this pattern was maintained until a gradual disassembly of the structures began at 120 min including SA exposure (Fig. 2B, *black line*; Suppl. Figure 3A). In contrast, SGs were detected in only 60% of primary MEFs after 30 min of SA stress. Surprisingly, there was no maintenance period in this cell type. Instead, SG rapidly disassembled after withdrawal of SA, such that SGs were detected in only 20% of fibroblasts 60 min post SA exposure (Fig. 2D, *black line*). Finally, the most surprising result was that cortical neurons were more resistant to SG formation, and SGs persisted longer. Treatment with 0.5 mM SA for 30 min, the gold-standard in the field, induced SGs in only 15% of the neurons, whereas treatment for 60 min was required to initiate robust SG formation (Fig. 2F*, black line*). (Note, 60 min SA treatment compromised the viability of MEFs and astrocyte cultures, precluding further analysis in these conditions.) This experiment suggests that primary cortical neurons are refractory to a certain level of stress exposure. However, once formed, SGs were maintained until 210 min, much longer than in other cell types, at which point there was a precipitous disassembly of SGs. Taken together, these results indicate there are detectable differences in the timing of SG formation and disassembly amongst different cell types relevant to ALS/FTD.

### TDP-43 regulates stress granule dynamics in primary cells exposed to oxidative stress

In transformed cells, TDP-43 depletion via siRNA impedes SG formation, reduces SG assembly (also referred to as secondary aggregation or coalescence)^11^ and accelerates SG resolution compared to control siRNA-treated cells^10^. Given the observations that different cell types have different kinetics of SG formation and disassembly, and our previous data that TDP-43 is central to SG dynamics in transformed cells, we questioned whether the involvement of TDP-43 also varies in cell types relevant to neurodegenerative diseases. In astrocytes in which nuclear TDP-43 expression was reduced to 39% of control levels (Fig. 2A), we observed a similar trend for the impact of TDP-43 on SG formation as assessed immediately following 30 min SA exposure, although the reduction did not reach statistical significance (Fig. 2B). However, SG disassembly was accelerated significantly in TDP-43-depleted astrocytes such that only 20% of cells demonstrated distinct SGs at 120 min, compared to 59% of siControl-treated primary astrocytes (Fig. 2B). In contrast, in MEFs where nuclear TDP-43 was reduced by 61% compared to siControl (Fig. 2C), the requirement of TDP-43 in SG formation was evident, with 64% of siControl-treated, but only 35% of siTDP-43-treated, fibroblasts demonstrating SGs at 30 min post-SA exposure (Fig. 2D). Similar results were obtained with a second independent siRNA for TDP-43 (Suppl. Figure 3). In cortical neurons, in which TDP-43 expression was modestly reduced by 40%, we did not observe a TDP-43-dependent effect on SG formation by this assay. With regards to disassembly, a minor defect was observed such that only 4% of TDP-43 depleted neurons contained SGs compared to 12% of control neurons at 270 min. Given the relatively weak suppression of endogenous TDP-43 levels by our experimental approach in cortical neurons, we suspect there is a certain threshold for TDP-43 level that must be surpassed for alterations in SG dynamics to manifest. Nonetheless, these data indicate that TDP-43 contributes to efficient SG dynamics in ALS/FTD-relevant cell types.

### Requirement of TDP-43 for SG coalescence is conserved in astrocytes and neurons

**Figure 3:**
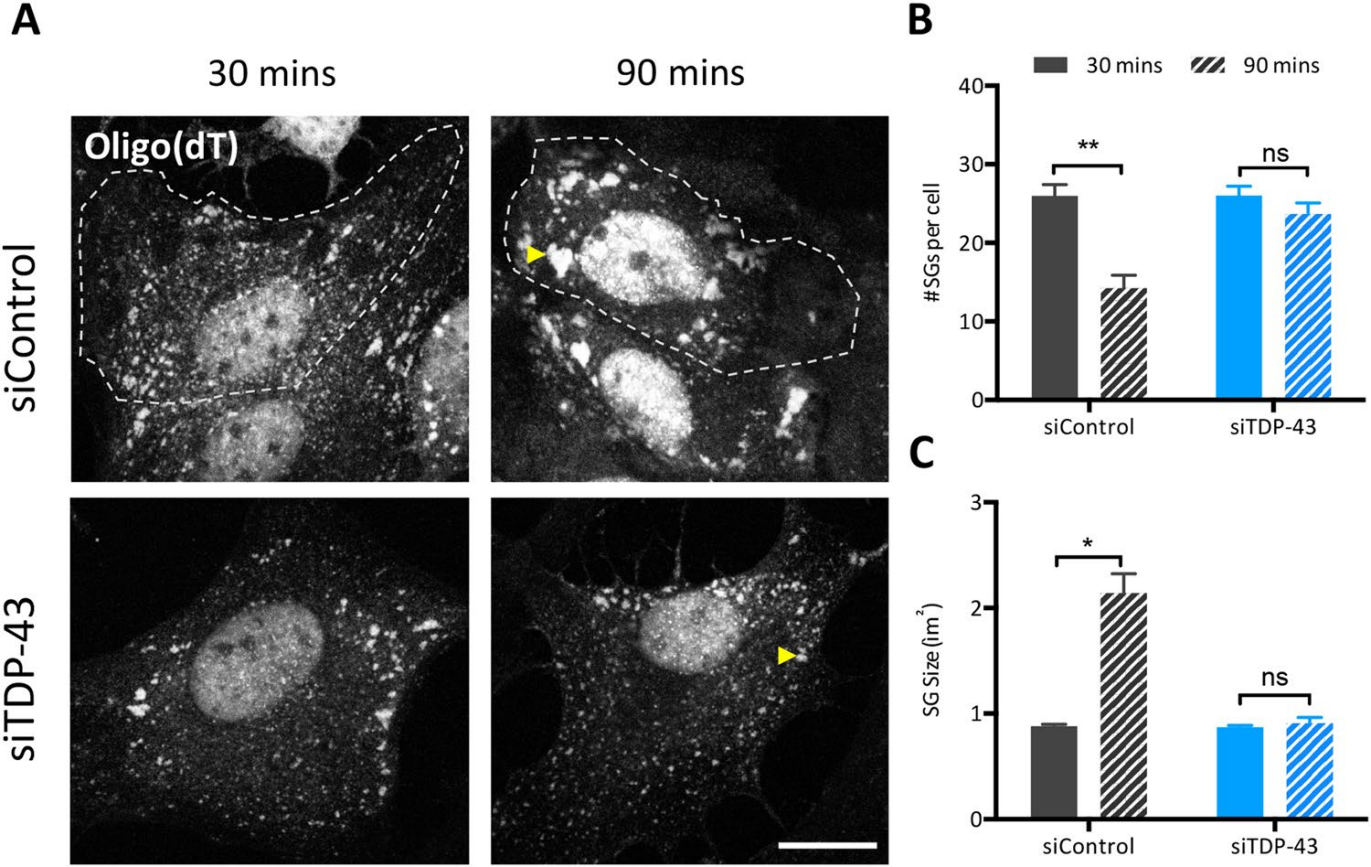
TDP-43 is required for SG assembly in astrocytes. (**A**) Representative images of primary astrocytes transfected with TDP-43 siRNA (siTDP-43 #2) and exposed to SA with cytoplasmic SGs marked with an oligo(dT) probe. Scale bar, 10 µm. (**B**) Number of SGs per cell and (**C**) size of individual SGs measured at the indicated time points post-SA exposure (n = 3, N = 10). Data is expressed as the mean ± SEM; Student *t* test *p < 0.05, **p < 0.01, ns: not significant.

We previously reported that TDP-43 is required for SG assembly in transformed cell lines^11^. Thus, the effect of TDP-43 knockdown by siRNA on stress granule dynamics was assessed in astrocytes and neurons. To measure SG coalescence, as indicated by SG size and number per cell, cells were assessed at high magnification over two time points following SA exposure. As expected, in control siRNA-treated astrocytes exposed to SA, the number of SGs per cell significantly decreased over time, from a mean of 26 SGs per cell at 30 min to 14 SGs per cell at 90 min (Fig. 3A,B, *black bars*) while the size of individual SGs increased about 1.2 μm^2^ between 30 and 90 min (Fig. 3C, *black bars*). In contrast, in siTDP-43 treated astrocytes selected based on diminished TDP-43 labelling (as measured by pixel intensity), individual SG size and number per cell remained unchanged when TDP-43 levels were lowered (Fig. 3B,C, *blue bars*). Similar results were obtained with a second independent siRNA for TDP-43 (Suppl. Figure 4A–C).

**Figure 4:**
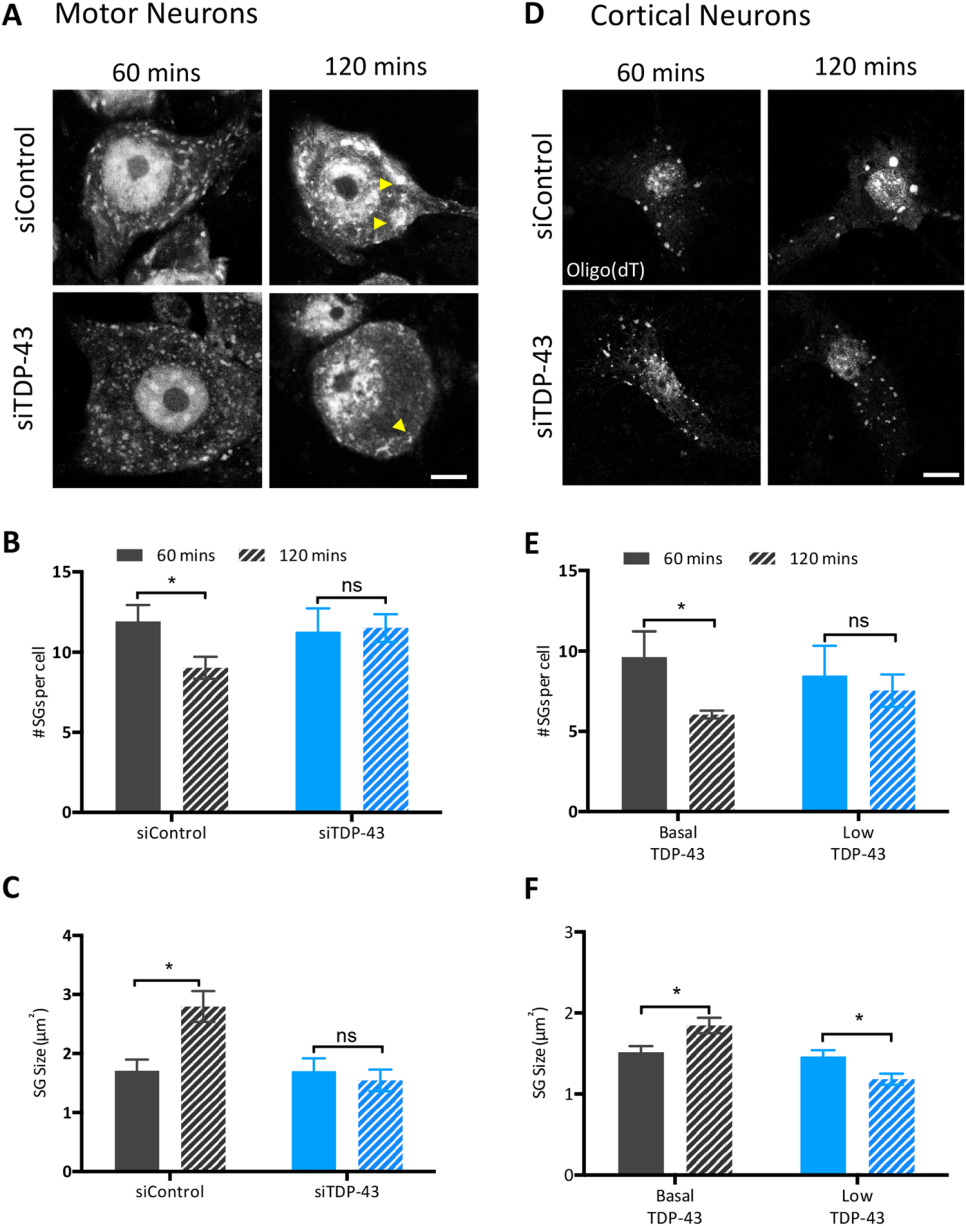
SG coalescence is mediated by TDP-43 in motor and cortical neurons. (**A**,**D**) Representative images of siRNA-microinjected motor neurons and siRNA transfected cortical neurons (both siTDP-43 #1) treated with SA and labelled for polyadenylated mRNA with oligo(dT). Arrowheads show SGs of interest. Scale bar, 10 µm. Number of SGs per cell and individual SG size in (**B**,**C**) motor neurons (n = 3, N = 10) and (**E**,**F**) cortical neurons (n = 4, N = 10) measured at the indicated time points post-SA treatment. Data is expressed as the mean ± SEM is shown; Student *t* test *p < 0.05, **p < 0.005, ns: not significant.

The same dependency on TDP-43 for SG coalescence was also observed in primary motor and cortical neurons. As visible in representative images (Fig. 4A, *shown by arrowhead*) siControl-injected motor neurons exposed to SA demonstrated the expected increased size of individual SGs of ~1 μm^2^ and a decrease in the number of SGs per cell from 60 to 120 min as in the previous experiments (Fig. 4B,C, *black bars*). In contrast, SG size and the number of SGs per cell did not change in motor neurons depleted of TDP-43 upon exposure to SA (Fig. 4B,C, *blue bars*). Similarly, SG coalescence was observed in cortical neurons expressing basal levels of TDP-43 following SA exposure (Fig. 4E,F, *black bars*), but not in TDP-43 siRNA-treated cortical neurons (Fig. 4E,F, *blue bars*). Specifically, neurons undergo a reduction in the number of SGs per cell over time, and a significant (albeit subtle) increase in individual SG size (Fig. 4E,F, *black bars*). These data were replicated with an independent TDP-43 siRNA (Suppl. Figure 4D–F). Note, although TDP-43 depleted cortical neurons do not demonstrate SG coalescence, an unexpected diminution of SG size at 120 mins was observed with one of the two TDP-43 siRNAs. Collectively, these studies demonstrate that TDP-43 is necessary for dynamic SG assembly in cell types relevant to ALS/FTD.

### TDP-43 is required for stress granule formation under osmotic stress

**Figure 5:**
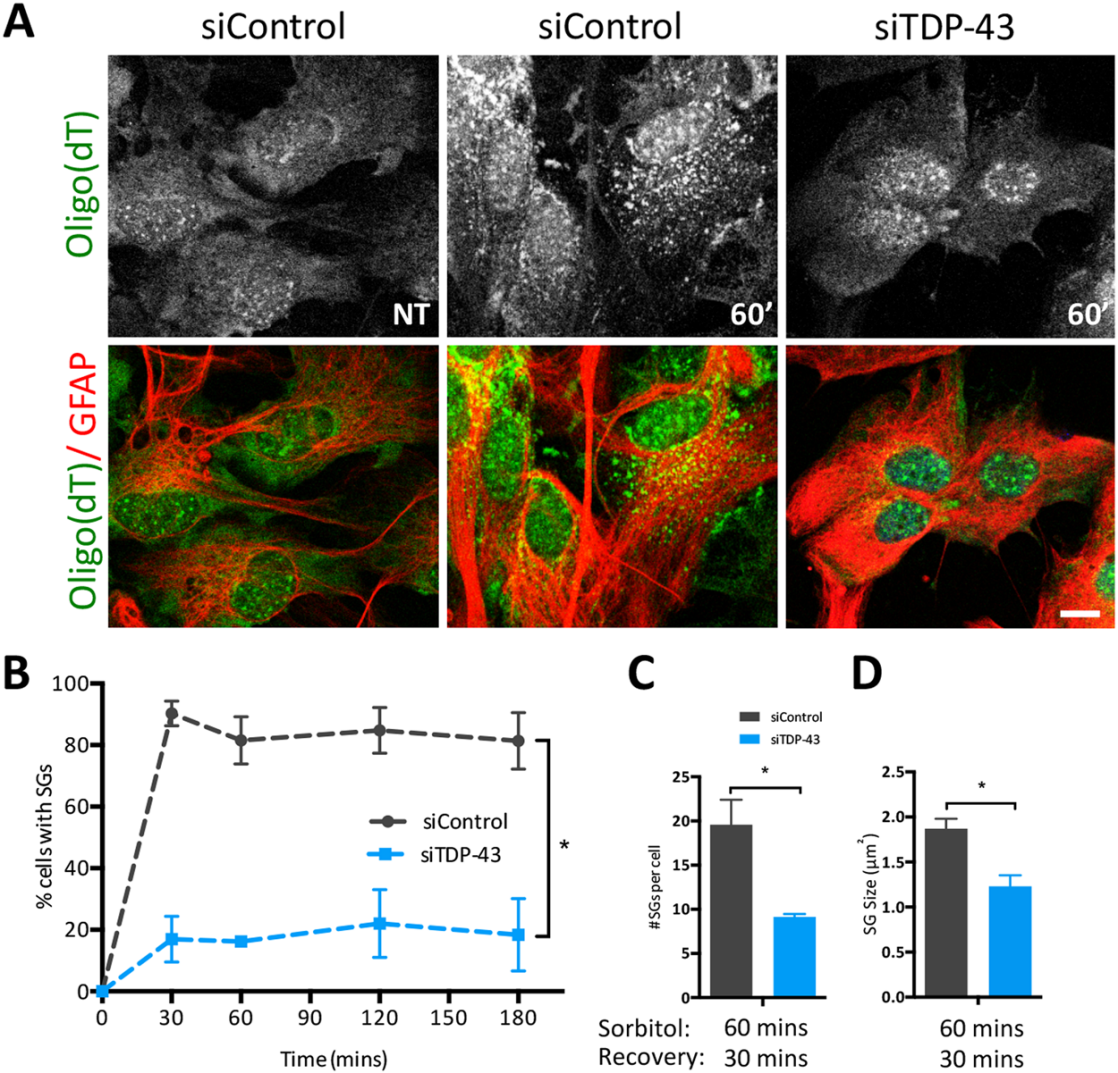
Astrocytes depleted of TDP-43 have abnormal SG kinetics and properties in response to hyperosmotic stress. (**A**) Representative images of siRNA transfected astrocytes (siTDP-43 #1) treated with 0.8 M D-Sorbitol. NT: non treated cells; 60′: 60 min of stress. Scale bar, 10 µm. (**B**) Number of cells with SGs at different time points during continual exposure to 0.8 M D-sorbitol (n = 3). (**C**) Number of SGs per cell and (**D**) size of individual SGs, induced with 0.8 M D-sorbitol for 60 min followed by 30 min of recovery (n = 3). Data is expressed as the mean ± SEM; Student *t* test *p < 0.05, **p < 0.005.

It is appreciated that SG composition can vary according to stress exposure^9,19,24,25^, and thus SG dynamics could also be variable. To determine if the effect of TDP-43 depletion on SG dynamics with SA-induced oxidative stress is stress-dependent or is a cell-intrinsic property, we exposed primary cortical neurons and astrocytes to hyperosmotic stress, a well published stress paradigm that has also been previously published to direct TDP-43 into SGs^19^. We tested a range of concentrations (0.4–1 M) of D-sorbitol and exposures for up to 3 h. Surprisingly, SGs were not observed in cortical neurons exposed to up to 1 M D-sorbitol (data not shown), while astrocytes robustly formed SGs in response to 0.8 M sorbitol stress, consistent with a previous report^19^. Under these conditions, SGs were very small. Indeed, although the SGs were near the diffraction limit and thus not able to be reliably measured, we inferred that the observed cytoplasmic puncta were SGs based on comparison to unstressed siControl astrocytes, which displayed a relatively disperse (*i.e*. non-granular) cytoplasmic mRNA pattern (Fig. 5A). Despite the inability to reliably measure individual SGs, analysis of the number of cells presenting with SGs revealed that the kinetics of SG assembly are severely impaired in astrocytes depleted of TDP-43. Specifically, only ~20% of astrocytes successfully formed SGs by 30 min compared to ~90% in siControl-treated cells, a phenomenon that persisted for the entire 180 min exposure (Fig. 5B). However, if cells were permitted to recover in normal growth media, individual SGs were larger, thus permitting an assessment of SG size and number. Specifically, at 30 min post D-sorbitol exposure, TDP-43 depleted astrocytes (Fig. 5C,D *blue bars*) had smaller and fewer SGs compared to siControl treated astrocytes (Fig. 5C,D *black bars*). These data establish the existence of cell type-specific responses to stress and further show that TDP-43 has variable relevance to SG dynamics in response to different stress conditions.

### Increased Aquaporin 4 levels in TDP-43 depleted astrocytes

**Figure 6:**
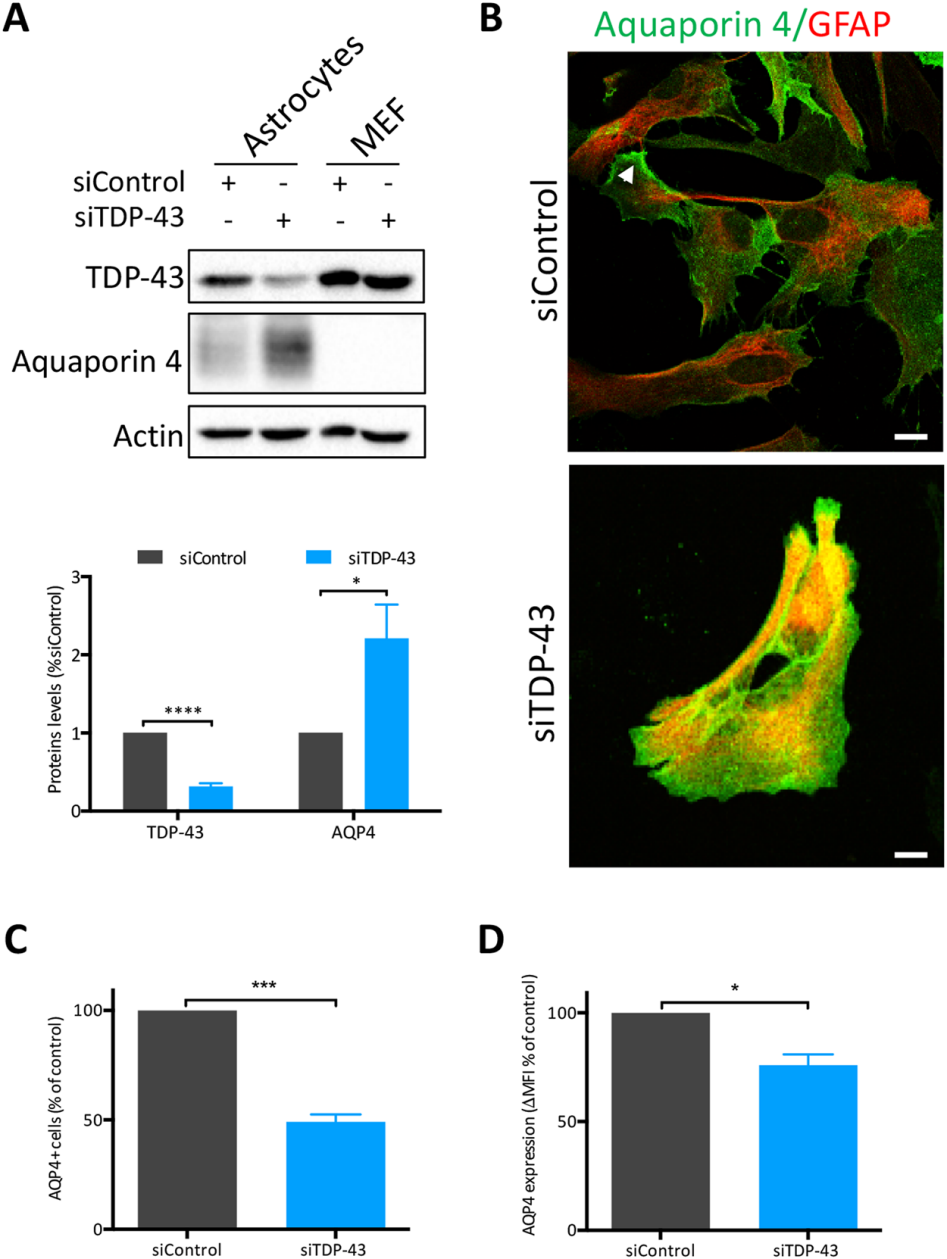
AQP4 is modulated by TDP-43. (**A**) Whole cell lysates of astrocytes and MEFs, treated with indicated siRNAs (siTDP-43 #1) were examined by immunoblotting for AQP4. Bands of interest were cropped from unmodified images, quantified via densitometry and normalized to Actin. (Uncropped blots are in Supplemental Material.) A representative experiment and quantification of 6 independent experiments are shown. (**B**) Immunofluorescence of siRNA-treated astrocytes showing localisation of AQP4 (green) on GFAP (red) positive cells. Arrowhead show astrocytic end feet. Scale bar, 10 µm. (**C**) AQP4 surface labelling of siRNA-treated astrocytes as assessed by flow cytometry (n = 4). (**D**) AQP4 median fluorescence intensity (ΔMFI over secondary control only) in astrocytes treated by siControl or siTDP-43 #1. Cells were analysed by flow cytometry (n = 4). Student *t* test *p < 0.05, ***p < 0.001, ****p < 0.0001.

Given the dramatic impact of TDP-43 on the SG response to osmotic imbalance, which is regulated by several different channel proteins, we investigated further. Aquaporins are a family of 13 bidirectional molecular water channels^26^ that are driven by osmotic gradients, of which two are mainly expressed in the CNS^27,28^. Of these, Aquaporin 4 (AQP4) is primarily expressed by astrocytes^29–31^ and has a prominent role in brain homeostasis and neuronal survival. AQP4 expression is elevated in select brain regions of patients affected with neurodegenerative diseases such as prion disease^32^, multiple sclerosis^27^ and ALS^26^, as well as in rodent models of mutant SOD1-mediated ALS^33^. In this study, by immunoblot, we found that AQP4 protein levels were increased 2-fold in TDP-43-depleted astrocytes compared to control siRNA-treated astrocytes and was absent from MEFs, as expected (Fig. 6A). A similar TDP-43-dependent elevation in AQP4 expression was also observed by immunofluorescence labelling; however, we noted that AQP4 was dispersed throughout the cytoplasm rather than at the end feet/processes, as observed in siControl cells (Fig. 6B, *arrowhead*). In attempt to determine if this increased expression results in more AQP4 channels at the plasma membrane, where it needs to be inserted to regulate osmotic balance, we performed surface labelling for AQP4 using non-permeabilized cells coupled with flow cytometric detection. Surprisingly, only 50% of TDP-43-depleted astrocytes had surface labelling for AQP4 relative to siControl astrocytes (Fig. 6C). In addition, in those siTDP-43 cells that were labelled, fluorescence intensity was reduced compared to control siRNA-treated astrocytes (Fig. 6D). Taken together, these data, verified with a second independent TDP-43 siRNA (Suppl. Figure 5), indicate that although total levels of AQP4 are elevated, it is not functional due to its absence at the cell surface.

### Aged neurons have compromised SG assembly

**Figure 7:**
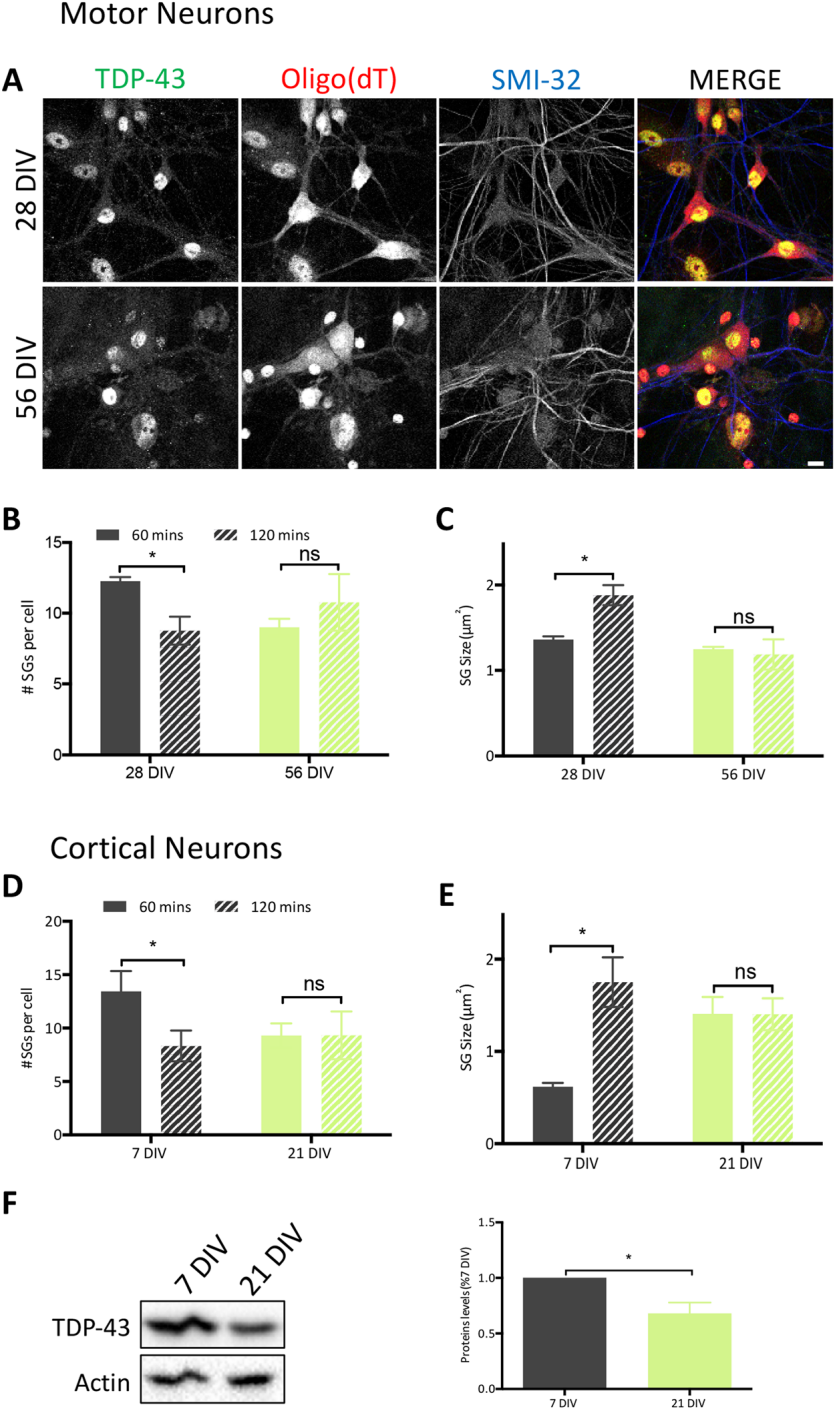
Aging negatively impacts SG assembly in neurons *in vitro*. (**A**) Representative micrographs of primary motor neurons aged in culture for 28 and 56 days and then subjected to 0.5 mM SA. SGs are marked with oligo(dT) (red) and neuronal processes marked with phosphorylated neurofilaments (SMI32, blue). Scale bar, 10 µm. Quantification of the number of SGs per cell and individual SG size in (**B**,**C**) motor neurons cultured for 28DIV and 56DIV (n = 3) and (**D**,**E**) cortical neurons cultured for 7DIV and 21DIV (n = 3). (**B**,**D**) Number of SGs per cell. (**C**,**E**) Size of individual SGs. Number and size were measured at two time points with ImageJ. (**F**) Whole cell lysates of cortical neurons at 7DIV and 21DIV immunoblotted for TDP-43. Bands of interest were cropped from unmodified images, quantified via densitometry and normalized to Actin. (Uncropped blots are in Supplemental Material.) A representative blot and the mean ± SEM of densitometric quantification of three independent experiments are shown. Student *t* test *p < 0.05.

As ALS and FTD are age-related diseases and SGs are considered to be a central component of disease pathogenesis, we investigated the impact of ‘aging’ of cortical and motor neurons on SG dynamics by prolonged culture, as a means to model aging *in vitro*^5,34^. To ensure that viability was maintained in these conditions, neurons were cultured in serum-free medium supplemented with B27, as described previously^34^. Motor neurons cultured according to this established protocol demonstrated robust axonal branches, the absence of obvious axonal swellings and/or retractions, and were morphologically indistinguishable between 28DIV (when these cultures are typically used for analysis) and 56DIV (Fig. 7A). In 28DIV motor neurons exposed to SA, there was a significant decrease in SG number and increased individual SG size between 60 and 120 min (Fig. 7B,C, *black bars*), consistent with what we observed in 28DIV siControl-injected motor neurons (Fig. 4B,C, *black bars*) indicating effective SG coalescence. However, in aged motor neurons (56DIV), SG coalescence did not proceed; size and number were unchanged over time (Fig. 7B,C, *green bars*). The same defect in coalescence was observed in cortical neurons cultured for 21DIV (an established *in vitro* aging paradigm for these neurons^34^) while SG assembly proceeded as expected in 7DIV cultures (Fig. 7D,E). Interestingly, TDP-43 levels were reduced by 32% in aged cortical neurons compared to 7DIV cortical neurons (Fig. 7F). Thus, these data suggest that aging (at least *in vitro*) compromises normal SG dynamics and may be linked to TDP-43 levels.

## Discussion

Environmental stress is considered to play a role in the phenotype of several neurodegenerative diseases, and SGs are a first line mechanism for survival following stress exposure. While much has been done in transformed cells, the molecular events in the cell types implicated in these diseases are not well documented. We used a stress model of primary motor and cortical neurons, as well as astrocytes, to study SG dynamics in primary cells relevant to ALS/FTD neurodegeneration.

We report differences in the morphology and kinetics of SGs formed in different cell types. Small SGs form quickly after arsenite stress and then rapidly disassemble in MEFs. In contrast, SGs formed in the majority of astrocytes within 30 min of arsenite treatment, followed by a plateau and subsequent decline at 150 min, indicative of SG disassembly similar to what we previously showed in SK-N-SH and HeLa cells^11^. Cortical neurons responded slowly to arsenite stress, with the majority of neurons displaying SGs only after 60 min of exposure. SG disassembly was noted after 300 min; this timeline of SG kinetics was twice as long in these neurons compared to astrocytes. While SG formation has been previously reported in primary neurons and glial cells^19,35^, this is the first study to detail the differences in SG assembly and disassembly kinetics in cell types relevant to neurodegenerative disease, namely neurons and astrocytes. Inclusions containing TDP-43 are characteristic of most cases of ALS and about half of all FTD cases. It has been proposed that SGs may serve to “seed” these inclusions^36^. One could speculate that the prolonged time required for SG assembly and disassembly may be relevant in this context and possibly contribute to neuronal vulnerability in these diseases. Indeed, persistent SGs due to delayed or failed disassembly have been reported where mutant forms of TDP-43 or other ALS-related genes have been transiently overexpressed in transformed cells^19,37^. In addition, SG disassembly is reportedly mediated by autophagy or chaperone-mediated protein degradation^38–42^, mechanisms which have been previously reported to become less efficient as neurons age^43,44^. That the kinetics of SG disassembly are protracted in neurons, coupled to the natural decline of these mechanisms intended to help dissipate SGs, may be highly relevant to the generation of the large cytoplasmic inclusions that characterize ALS/FTD, and are rich in RNA binding proteins beyond just TDP-43.

Second, we demonstrate the importance of TDP-43 in SG assembly and disassembly in neurons and astrocytes. Specifically, reducing the levels of TDP-43 resulted in an acceleration of SG disassembly in astrocytes and cortical neurons. We attribute the more modest effect in cortical neurons to a less efficient depletion of TDP-43 levels. Interestingly, SG coalescence was impaired by TDP-43 depletion in all cell types examined and reaffirms the important role of TDP-43 in regulating this essential cell stress response mechanism previously documented in transformed cells^11^. We have previously shown that SG coalescence is relevant to SG interactions with processing bodies and the protection of polyadenylated mRNA following stress exposure^45^. Thus, we speculate that reduced nuclear TDP-43 levels would have negative consequences on these aspects of RNA granule homeostasis in neurons and astrocytes as well. This could, in turn, impact RNA homeostasis in general, possibly leading to a complex dysregulation of both transcript levels/stability and miRNA processing.

In primary astrocytes, we discovered that TDP-43 is critically required for proper SG formation in hyperosmotic conditions. Specifically, following assembly, the SGs formed in TDP-43 depleted cells are smaller compared to control siRNA-treated astrocytes. We postulated that the glial-relevant water channel AQP4 may be impacted by TDP-43. Indeed, we observed that AQP4 was less efficiently integrated into the plasma membrane despite a robust TDP-43-dependent up-regulation. AQP4 has been previously reported to be up-regulated in mouse models expressing mutant SOD1^33,46^ as well as in patients^27^, and a loss of polarity of the protein has been reported^46^. TDP-43-mediated regulation of AQP4 expression and trafficking has not previously been reported and may represent a novel mechanism for further exploration given that astrocytes are known to play a contributing role to neurodegeneration^47,48^.

Finally, we explored the impact of aging on SG formation in response to stress by subjecting neurons to long-term culture conditions, an established *in vitro* model of aging. Our data indicate that although aged motor and cortical neurons form SGs in response to oxidative stress, they do not coalesce as expected. With the important caveat that aging cultures *in vitro* is not equivalent to *in vivo* aging, our findings suggest the possibility of a disturbance of RNA granule dynamics during normal aging. In addition, an unexpected observation was that TDP-43 protein levels were diminished in the aged cultures. Aging has long been considered as a risk factor for ALS and FTD^20^. Indeed, current models propose that these diseases arise due to “two-hits”, genetic susceptibility and a second trigger such as external stress or aging. Although we appreciate that these results are *in vitro*, it is tempting to speculate that aging could be a modifier of SG dynamics and thus may have relevance to the initiation and/or progression of age-related neurodegenerative diseases. Taken together, our data suggest that the cell and stress specificity in SGs dynamics and the impact of aging may be contributing factors to neuronal vulnerability in ALS and FTD cases with TDP-43 inclusions. A better understanding of the molecular mechanisms surrounding this stress response in a cell specific manner will be essential for future ALS/FTD therapeutic strategies aimed at modulating stress granule dynamics.

## Material and Methods

### Preparation and transfection of mouse primary cells

The use of animals and all procedures were performed according to the guidelines of the Canadian Council on Animal Care and were approved by the CRCHUM Institutional Committee for the Protection of Animals, and MNI/McGill / Animal Care Committee. Primary cultures of dissociated spinal cord along with dorsal root ganglia (DRG) were prepared as described^49^, from embryonic day 13 (E13) CD1 mouse embryos (Charles River Laboratories, St. Constant, QC, Canada). After dissociation in trypsin, cells were plated at a density of ~450,000 per well in 12-well dishes (Greiner Bio-One) containing round glass 18-mm coverslips (Thermo Fisher Canada) coated with poly-D-lysine (Sigma-Aldrich, Oakville, ON, Canada) plus Matrigel basement membrane matrix (Corning, Corning, NY, USA). Cultures were maintained in minimum essential medium enriched with 5 mg/ml glucose and supplemented with 1.3% horse serum (Invitrogen), 10 μg/ml bovine serum albumin, 26 ng/ml selenium, 20 μg/ml triiodothyronine, 10 μg/ml insulin, 32 μg/ml putrescine, 9.1 ng/ml hydrocortisone, 13 ng/ml progesterone (Sigma-Aldrich), 200 μg/ml apo-transferrin (US Biological), and 10 ng/ml nerve growth factor (UBI Life Sciences). On days 4–6, cultures were treated with 1.4 μg/ml cytosine-β-D-arabinoside (Calbiochem, San Diego, CA, USA) to minimize growth of non-neuronal cells. The cultures were maintained at 37 °C in 5% CO_2_. Cultures were used at 21 or 56 days post-plating, as indicated in the figure legends. At this age *in vitro*, motor neurons are distinguishable from other neuronal types and glia in the culture because they develop and differentiate to resemble their counterparts in the intact spinal cord, both morphologically and by expression of biological markers. They have large cell bodies (>20 µm in diameter) and dendritic trees, and express choline acetyltransferase, glutamate receptors, and neurofilament proteins^50–52^.

Primary cortical neurons were prepared from E18.5 C57BL/6 mouse embryos (Charles River Laboratories, Kingston, NY, USA), as previously published^53^. Briefly, cerebral cortices were trypsinized (0.025%, 37 °C, 20 min), and then treated with trypsin inhibitor (0.52 mg/ml, Sigma T9003) and DNAse (1.7 KU, Sigma D5025). Neurons were dissociated by trituration with a Pasteur pipette. Cells were then plated on glass coverslips coated with polylysine and laminin (Sigma Aldrich) and maintained in Neurobasal medium (Invitrogen) supplemented with 0.0025% Glutamax (Invitrogen) and 0.02% B27 (Invitrogen). Cultures were used five days after plating, or as indicated in the figure legend.

Primary astrocytes were cultured from C57BL/6 pups (P0–P3) bred in house from stock obtained from Charles River Laboratories (Kingston, NY, USA). Cortices were dissociated with 18 G and 21 G needles and cells were grown in Dulbecco’s modified Eagle medium (DMEM, Invitrogen), supplemented with 10% FBS, 1% glutamine and 1% Penicillin-Streptomycin (Sigma) on poly-L-ornithine (Sigma) coated plates and coverslips. Cells were used at confluence (~14 DIV).

Primary mouse embryonic fibroblasts (MEFs) were prepared from E12.5 C57BL/6 embryos (Charles River Laboratories Kingston, NY, USA). Briefly, the entire body except the liver was treated with trypsin (0.025%, 37 °C, 30 mins). The reaction was stopped with 10% FBS supplemented high Glucose DMEM and the cells were dissociated mechanically and plated in DMEM (Invitrogen) supplemented with 10% FBS, 1% glutamine and 1% Penicillin-Streptomycin (Sigma).

Astrocytes and MEFs were transfected at 60–70% confluence with 40 pmol of custom siRNAs using Lipofectamine 2000 (Invitrogen), according to the manufacturer’s instructions and collected after 72 h. siRNA sequences used were: mouse TDP-43 #1, 5′-AAGCAAAGCCCAGACGAGCCUUUGA-3′; mouse TDP-43 #2 (MSS214149, Thermo Fisher Scientific): 5′-GCAAUCUGGUAUAUGUUGUCAACUA-3′; and the negative control low GC siRNA (#12935–200; Invitrogen). Cortical neurons were transfected at 7 DIV for 5 days with 5 pmol (per 18 mm coverslip) of mouse siTDP-43.

### Microinjection of mouse primary motor neurons

Motor neurons in mature spinal cord cultures do not transfect; thus, plasmids are expressed by intranuclear microinjection. The protocol was performed as previously published^50^. Specifically, cultures were bathed in EMEM without sodium bicarbonate, titrated to pH 7.2. Solutions of Tris-EDTA (5 mM Tris and 0.5 mM EDTA), pH 7.2, containing 125 pmol of siRNA (negative control low GC and mouse TDP-43) plus 70 kDa dextran (25 mg/ml; Molecular Probes Inc., Eugene, OR) were clarified by centrifugation at 11,000 × g for 15 min and microinjected into neuronal nuclei using glass microcapillaries (World Precision Instruments) coupled with a microinjector (composed of InjectMan NI 2 and FemtoJet; Eppendorf). After completion of the injection, cultures were returned to the incubator in culture medium plus 0.75% gentamicin (Gibco, Burlington ON).

### Induction of stress granules

To induce SGs, cells were treated with 0.5 mM SA (Sigma-Aldrich) for the indicated times at 37 °C. Recovery was initiated by changing the media. For osmotic stress, astrocytes were treated with 0.8M-1M D-Sorbitol (Sigma) for indicated times. Cover slips were collected and fixed with 1% paraformaldehyde (PFA) at 30, 60, 120, and 180 min after the start of the stress.

### Fluorescence *in situ* hybridization and immunofluorescence

Cells grown on coverslips were fixed in 1% PFA in PBS and then permeabilized with 0.1% Triton X-100 in 2X saline sodium citrate (SSC) for 15 mins, then washed with 1 M Tris, pH 8 for 5 mins. Coverslips were blocked with 0.0005% BSA, 10 mg/ml yeast RNA diluted in 2X SSC, washed with 1 M Tris, pH 8 for 5 mins, and incubated with hybridization buffer (1.3 ng FITC or Cy3 labelled oligo(dT) probe, 0.005% BSA, 1 mg/ml yeast RNA, 10% dextran sulfate, and 25% formamide, diluted in 2X SSC) for 1 h in a humid chamber. Coverslips were subsequently washed twice with 4X SSC for 5 min and once with 2X SSC for 5 mins Coverslips where incubated for 1 h with primary antibody: rabbit TDP-43 (1/200, Proteintech 10782–2-AP), and when indicated with SMI32R (1/2000, Covance 801701), GFAP (1/20000, Abcam ab4674; or mouse anti-GFAP conjugated with Cy3, Sigma C9205), HuR (1/200, Millipore 07–468), CAPRIN1 (1/200, Proteintech 15112–1-AP). After washing twice with 4X SSC for 5 min and once with 2X SSC for 5 min, labelling was visualized with fluorescently conjugated secondary antibodies against the desired species (Jackson ImmunoResearch). Coverslips were washed twice with 4X SSC, once with 2X SSC, and then mounted with ProLong Antifade reagent (Invitrogen). Images were collected using 40X (1.25 NA) and 63X oil (1.7 NA) objective lenses on a confocal microscope (SP5; Leica) equipped with LAS AF software (Leica) for acquisition. Total cellular fluorescence intensity of the oligo(dT) probe and TDP-43 was determined using Photoshop CS4 (Adobe). Discrimination of TDP-43 levels was based on measurements of TDP-43 pixel intensity in controls/at baseline in the accompanying experiment. Importantly, all images were acquired with the same settings.

### Quantification of SGs number and size

SGs parameters were quantified using ImageJ, as previously published^10,11^. Briefly, to assess SG kinetics at different time points, SGs were identified by oligo(dT), and cells were scored as positive when they had at least two foci. A minimum of 70-100 cells were counted per time point, per condition and per experiment. For SG number and size, we used automatic recognition by ImageJ (function: Analyse particles) using the following parameters: all SGs ranging from 0.2 to 15 µm^2^ in 10 randomly selected cells per condition in each experiment.

### Western Blot

As we previously published^11^, cells were collected by scraping in ice-cold PBS. Lysis was performed with RIPA buffer (150 mM NaCl, 50 mM Tris pH 7.4, 1% Triton X-100, 0.1% SDS, 1% sodium deoxycholate, and protease inhibitors), incubated 10 min on ice, 10 min at room temperature then centrifuged 10 min at 13500 *g*. The collected supernatants were quantified with the BCA Protein Assay Kit (Thermo Scientific, Waltham, MA, USA). 20 µg of lysates were loaded for standard SDS-PAGE. The antibodies used were: rabbit anti-TDP-43 (1:10 000; 10782–2-AP Proteintech, Chicago, IL, USA), rabbit anti-AQP4 (1:2000; 16473–1-AP Proteintech), and mouse anti-Actin (1:200 000; 69100 MP Biomedicals, Santa Ana, CA, USA) as a loading control. Blots were visualized with peroxidase-conjugated secondary antibodies and ECL Western Blotting Substrate (Pierce, Waltham, MA, USA). Images were acquired using a range of acquisition times using the BIO-RAD ChemiDoc MP imaging system, and then exported as tifs via the Image Lab application without any manipulation. Chosen images were those just below overexposure (easily seen by the red pixels on the image). Densitometry was then performed with Adobe PhotoShop. No post-acquisition modifications were made.

### Flow Cytometry

Extracellular staining of AQP4 was performed as previously described^54^. Briefly, cells were detached and labelled for surface AQP4 with primary antibody (Proteintech 16473–1-AP) and secondary antibody (Jackson ImmunoResearch). Note, the recognized epitope is resistant to trypsin digestion. Non-specific background staining was assessed using an appropriate fluorochrome-matched secondary antibody. Cells were processed the same day for analysis on a BD LSR II flow cytometer and data was analyzed using FACSDiva (BD Bioscience).

### Statistics

Data were compared via two-tailed Student *t* test as indicated in the figure legends, with statistical significance established at p < 0.05.

## Electronic supplementary material

Supplementary information
