# Supplementary material for: TDP-43 regulation of stress granule dynamics in neurodegenerative disease-relevant cell types
Source: Sci Rep. 2018 May 15;8:7551. doi: 10.1038/s41598-018-25767-0 (PMC5953947; doi:10.1038/s41598-018-25767-0)
Supplement: Supplementary file 1 — Supplementary information [file 41598_2018_25767_MOESM1_ESM.pdf]

TDP-43 regulation of stress granule dynamics in neurodegenerative disease-relevant cell types

Yousra Khalfallah[1,3], Rachel Kuta[4], Camille Grasmuck[2,3], Alexandre Prat[2,3], Heather Durham[4] and Christine Vande Velde[2,3]*

Departments of [1]Biochemistry and Molecular Medicine and [2]Neurosciences, Université de Montréal, [3]Centre Hospitalier de l'Université de Montréal (CHUM) Research Centre [4]Montreal Neurological Institute and Department of Neurology/Neurosurgery, McGill University, Montréal, QC, Canada.

***Corresponding author:**

Christine Vande Velde, Ph.D.
Department of Neurosciences, Universite de Montreal
CRCHUM-Tour Viger
900, rue Saint-Denis, R09.442
Montreal, QC, CANADA H2X 0A9

Email: c.vande.velde@umontreal.ca
Phone: 514-890-8000 poste 28832

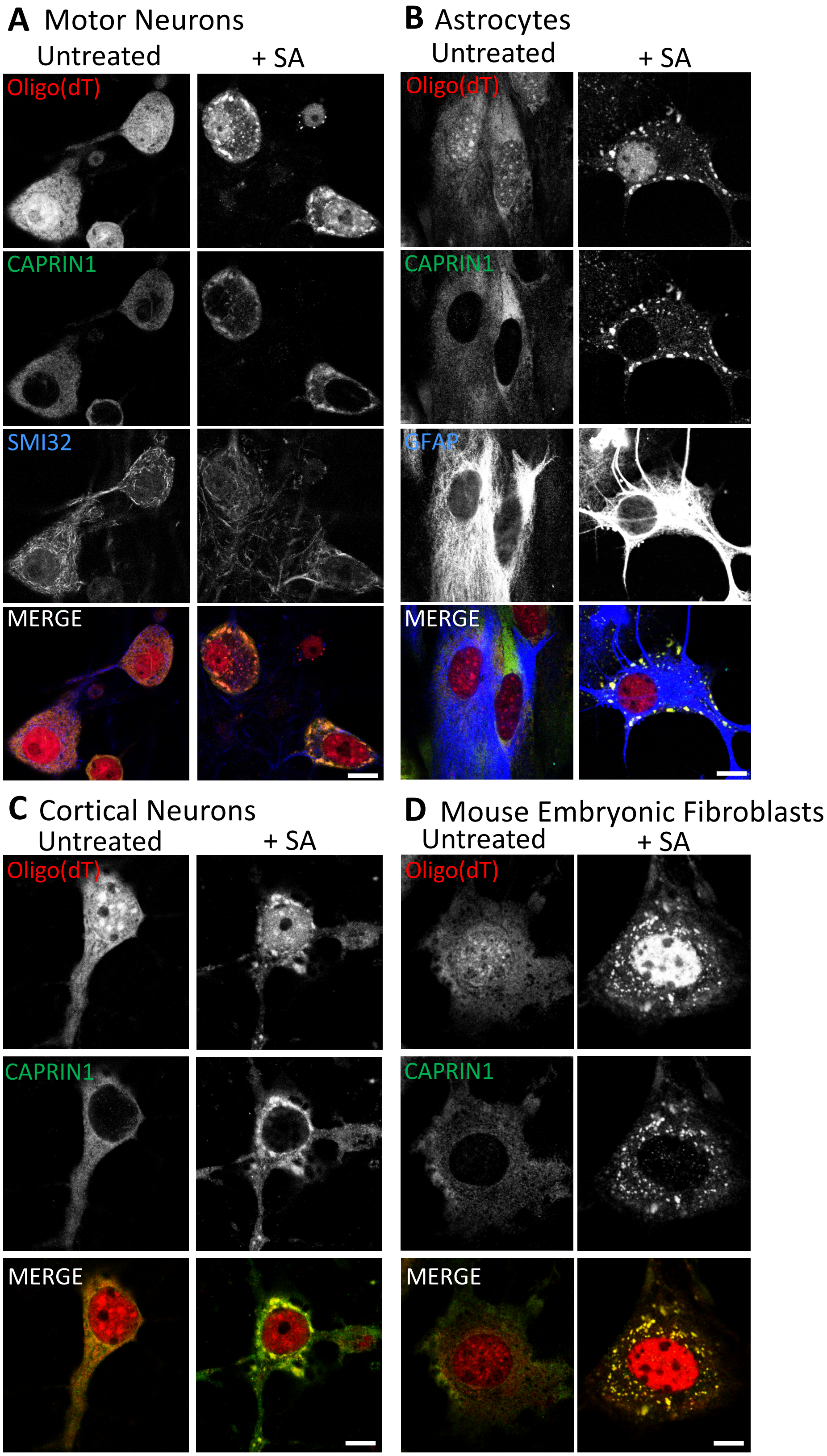

Supplementary figure 1. Differences in SG morphology amongst cell types. Primary cultures of (A) motor neurons, (B) astrocytes, (C) cortical neurons and (D) mouse embryonic fibroblasts treated (or not) with 0.5 mM of sodium arsenite (+SA). Cytoplasmic SGs were co-labelled with an oligo(dT) probe, to track polyadenylated mRNA, and an antibody against CAPRIN1 (a known SG marker). Note, motor and cortical neurons were treated for 60 min while astrocytes and fibroblasts were treated with 30 min. Scale bar, 10 μm.

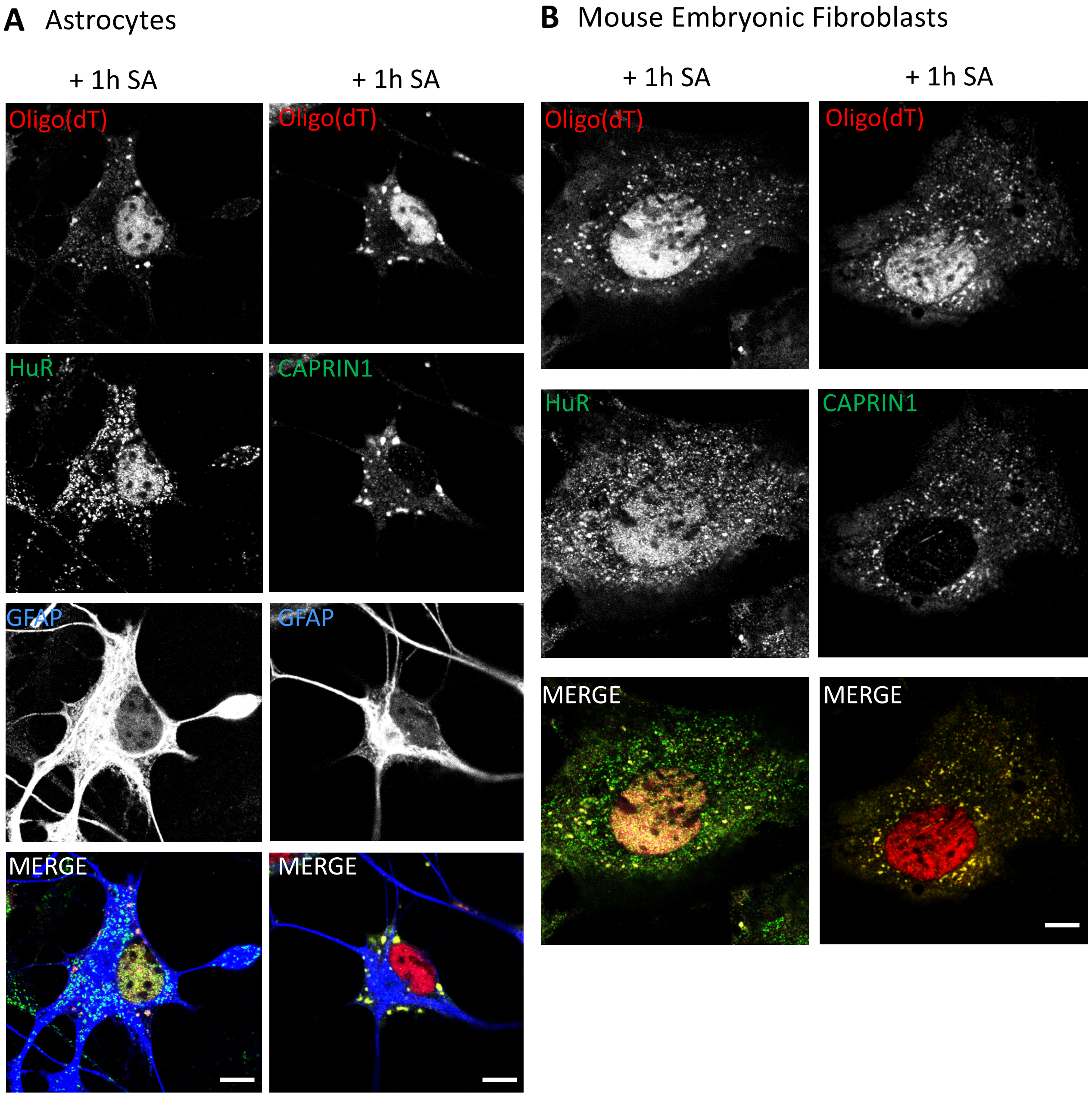

Supplementary figure 2. SG morphology after 60 min SA treatment. Primary cultures of (A) astrocytes, (B) mouse embryonic fibroblasts treated (or not) for 60 min with 0.5 mM of sodium arsenite (+SA). Cytoplasmic SGs were co-labelled with an oligo(dT) probe, to track polyadenylated mRNA, and an antibody against HuR or CAPRIN1. Scale bar, 10 µm.

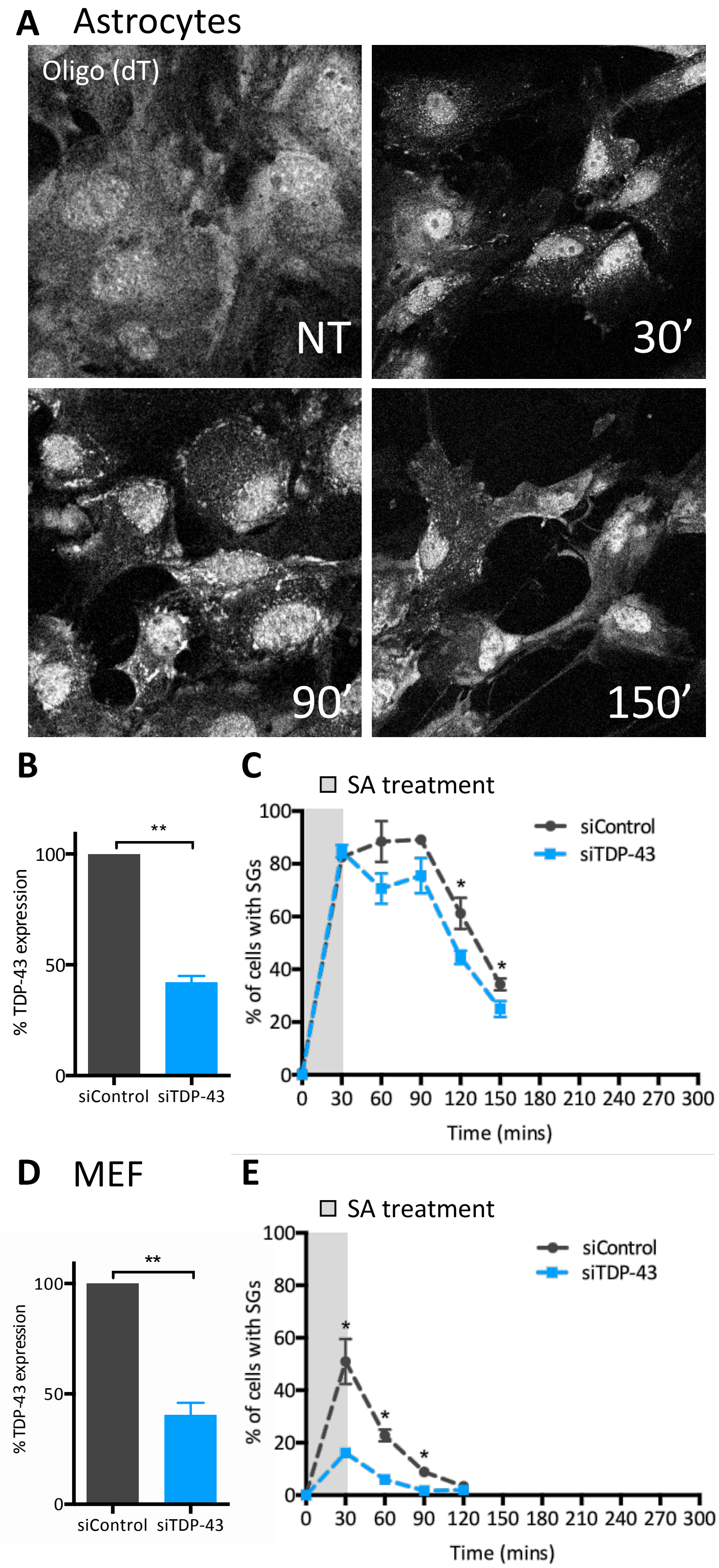

Supplementary figure 3. SG kinetics vary according to cell type and are modulated by TDP-43. (A) Representative images of primary astrocytes treated with SA with cytoplasmic SGs marked with an oligo(dT) probe. NT: non treated cells; 30’: 30 mins of stress; 90’: 90 mins time point; 150’: 150 mins time point. Scale bar, 10 μm. (B, C) Primary astrocytes (n=3, average N=100 per time point), (D, E) mouse embryonic fibroblasts (MEF) (n=3, average N=100 per time point), were transfected with siRNA (siTDP-43 #2) and subjected to 0.5 mM SA. (B, D) TDP-43 expression levels expressed relative to control cultures, as determined by measurement of TDP-43 signal intensity. (C, E) Percentage of cells displaying SGs at different time points following SA exposure. Data of 3 independent experiments are expressed as the mean ± SEM; Student *t* test $^*p < 0.05$, $^{**}p < 0.01$.

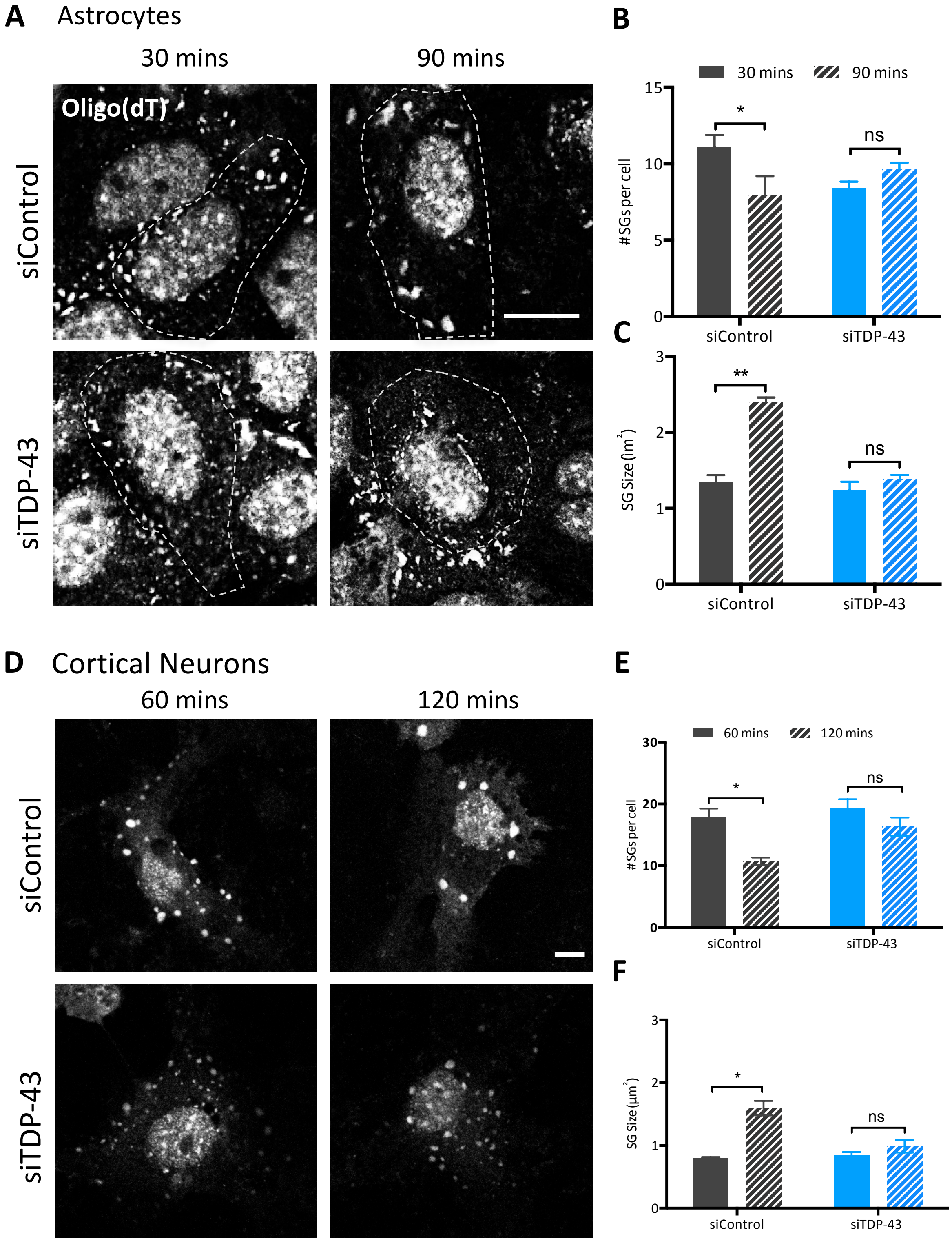

Supplementary figure 4. TDP-43 is required for SG assembly in astrocytes and cortical neurons. (A, D) Representative images of primary astrocytes transfected with TDP-43 siRNA (siTDP-43 #1) and primary cortical neurons transfected with TDP-43 siRNA (siTDP-43 #2), exposed to SA with cytoplasmic SGs marked with an oligo(dT) probe. Scale bar, 10 µm. (B, E) Number of SGs per cell and (C, F) size of individual SGs measured at the indicated time points post-SA exposure (n=3, N=10). Data is expressed as the mean ± SEM; Student *t* test *p < 0.05, **p <0.005, ns: not significant.

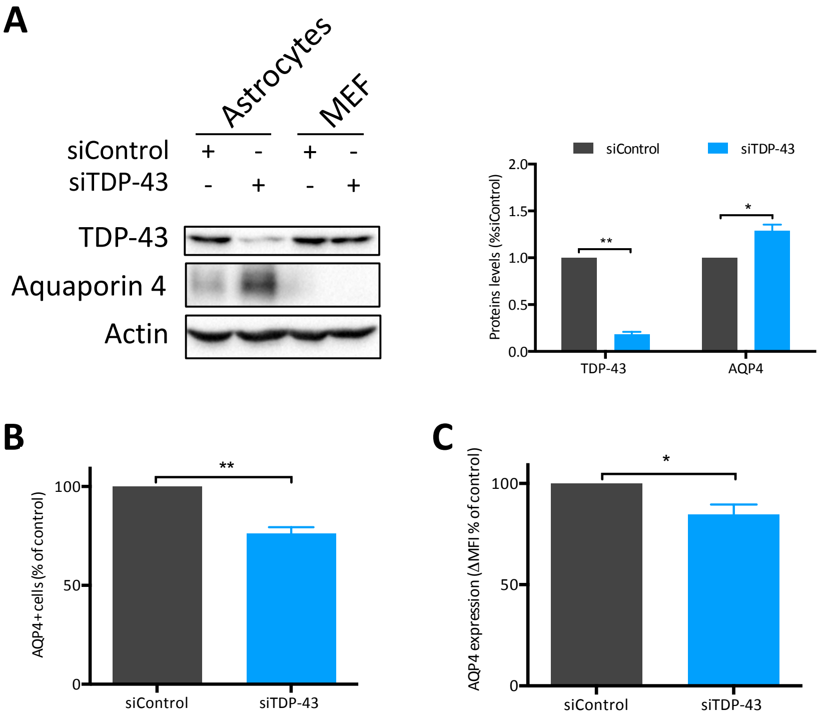

Supplementary figure 5. AQP4 is modulated by TDP-43. (A) Whole cell lysates of astrocytes and MEFs, treated with indicated siRNAs (siTDP-43 #2) were examined by immunoblotting for AQP4. Bands of interest were cropped from unmodified images, quantified via densitometry and normalized to Actin. (Uncropped blots are in Supplemental Material) A representative experiment and quantification of 3 independent experiments are shown. Student *t* test ***p < 0.001, **p < 0.005, *p < 0.05. (B) AQP4 surface labelling of siRNA-treated (siTDP-43 #2) astrocytes as assessed by flow cytometry (n=3). (C) AQP4 median fluorescence intensity (ΔMFI over control secondary only) in astrocytes treated by siControl or siTDP-43 (siTDP-43 #2). Cells were analysed by flow cytometry n=3. Student *t* test *p < 0.05, **p < 0.005.

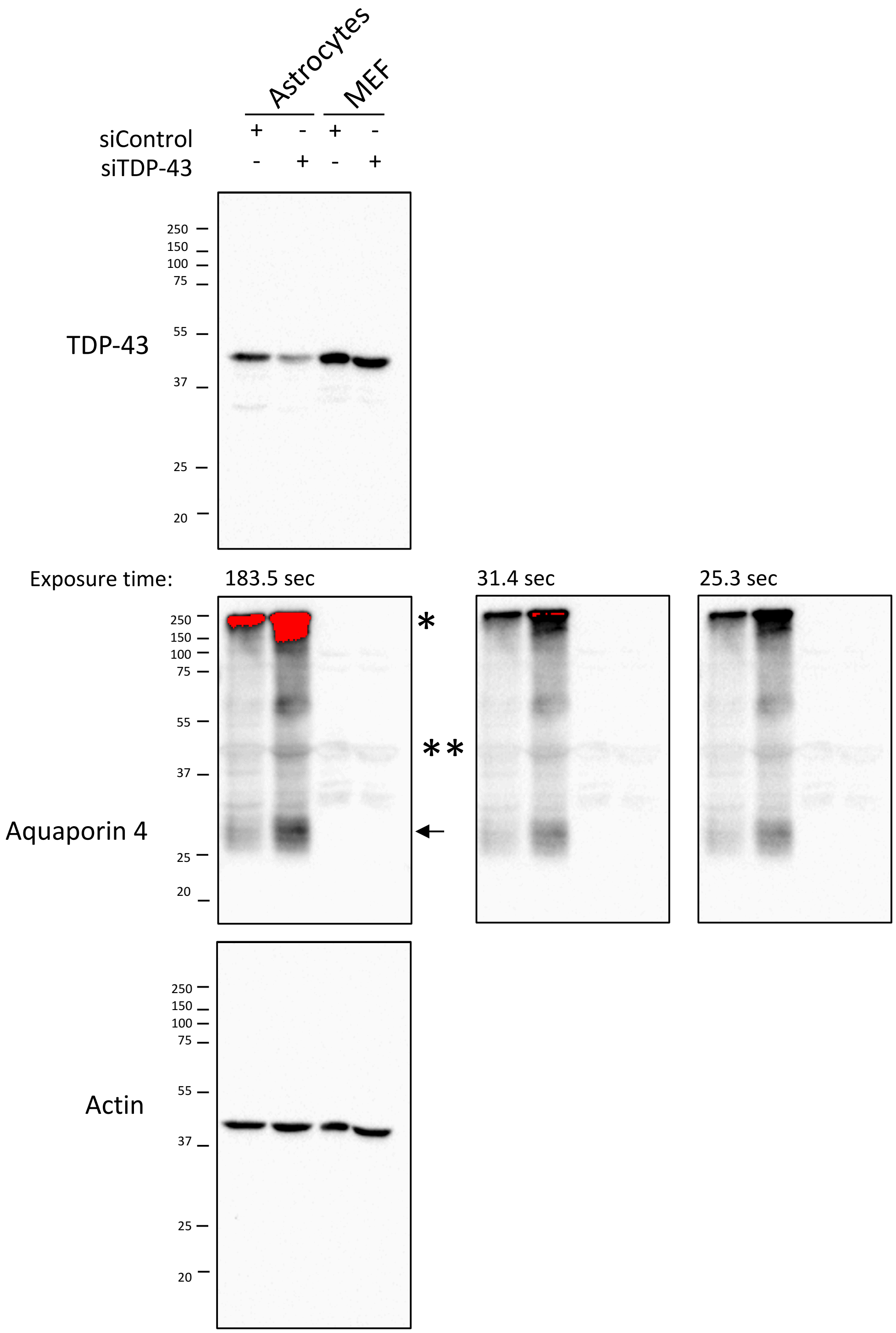

Supplementary material 1. TDP-43 siRNA increases AQP4. This figure contains uncropped western blots from which Figure 6 is derived. Whole cell lysates from astrocytes and MEFs treated with indicated siRNAs were probed for TDP-43, AQP4 and Actin on the same membrane. * indicates the tetramer of AQP4 which was insufficiently denatured to enter the gel. Images are shown with different exposure time for this overexposed band. ** indicates background band from TDP-43 antibody which was applied prior to AQP4. Arrow indicates AQP4 monomer at 32-34 kDa, as expected.

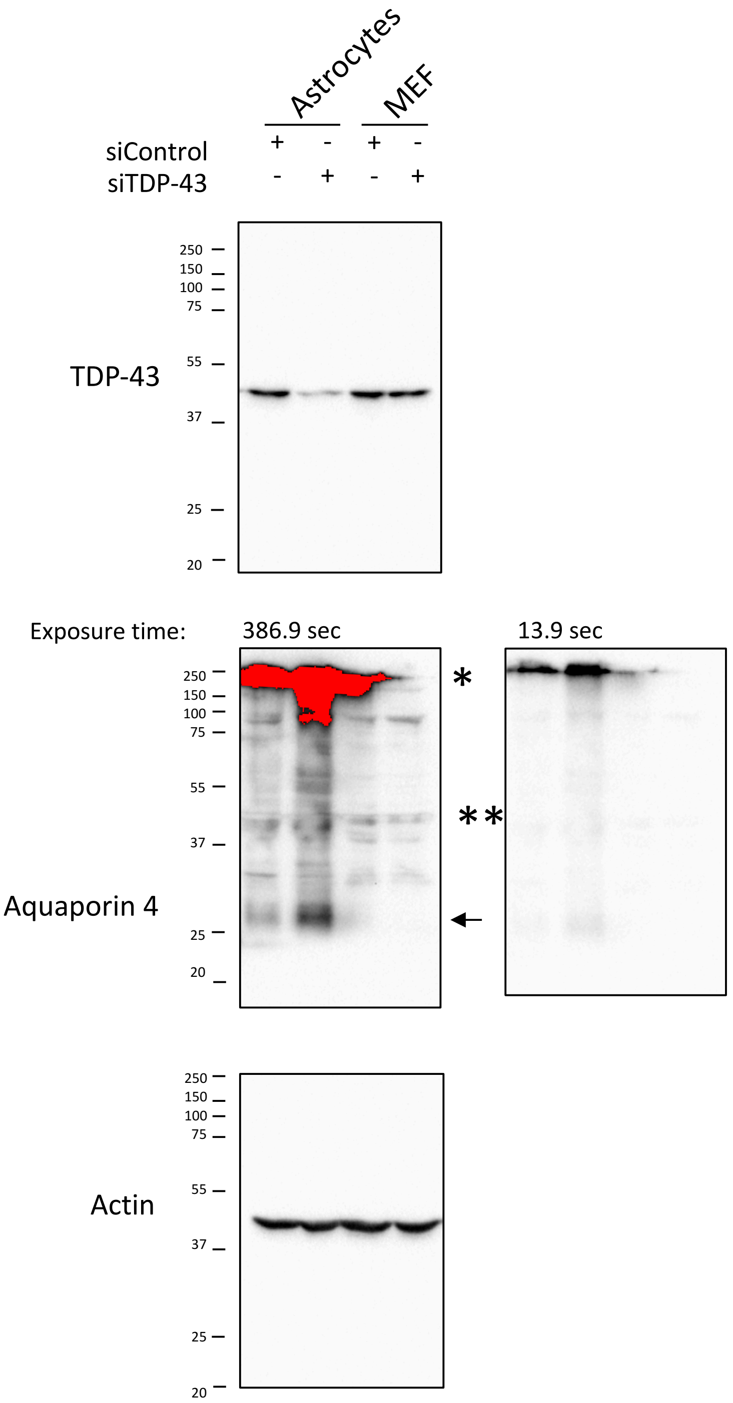

Supplementary material 2. TDP-43 siRNA 2 increases AQP4. This figure contains uncropped western blots from which supplementary Figure 5 is derived. Whole cell lysates from astrocytes and MEFs treated with indicated siRNAs (siTDP-43 #2) were probed for TDP-43, AQP4 and Actin on the same membrane. * indicates the tetramer of AQP4 which was insufficiently denatured to enter the gel. Images are shown with different exposure time for this overexposed band. ** indicates background band from TDP-43 antibody which was applied prior to AQP4. Arrow indicates AQP4 monomer at 32-34 kDa, as expected.

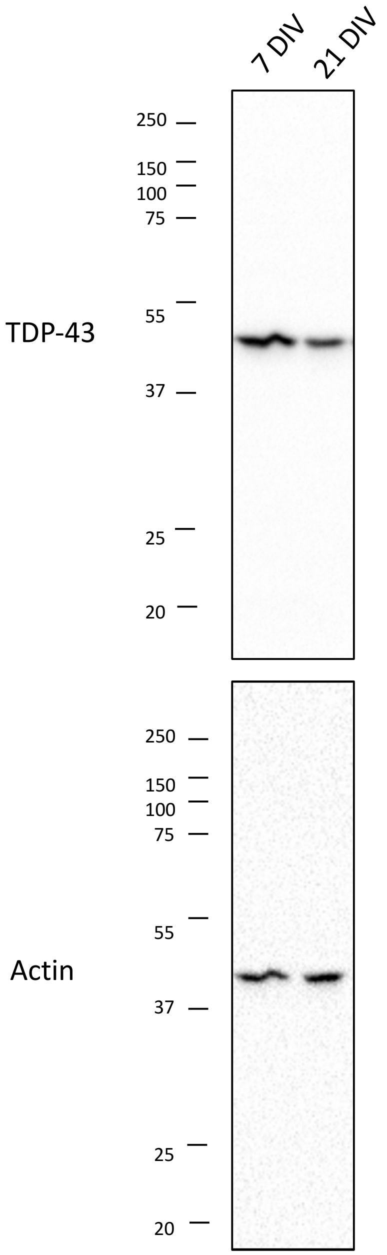

Supplementary material 3. TDP-43 levels are decreased in aged neurons *in vitro*. Uncropped films from which Figure 7F is derived. Whole cell lysates of 7DIV and 21DIV cortical neurons were immunoblotted for TDP-43 and Actin on the same membrane.
